# Microproteins in Metabolism

**DOI:** 10.3390/cells14120859

**Published:** 2025-06-07

**Authors:** Caris A. Wadding-Lee, Catherine A. Makarewich

**Affiliations:** 1 Division of Molecular Cardiovascular Biology, The Heart Institute, Cincinnati Children’s Hospital Medical Center, Cincinnati, OH 45229, USA; 2 Department of Pediatrics, University of Cincinnati College of Medicine, Cincinnati, OH 45229, USA

**Keywords:** microprotein, metabolism, mitochondrial function, small open reading frame

## Abstract

Metabolism is a complex network of biochemical pathways that break down macromolecules to produce energy essential for cellular function. Disruptions in metabolic homeostasis are closely linked to noncommunicable diseases (NCDs) such as cardiovascular disease, type 2 diabetes, and cancer, which are leading causes of death worldwide. Many NCD-associated conditions, including obesity and insulin resistance, stem from metabolic dysfunction, and current therapies often fall short in preventing disease progression, highlighting the need for novel therapeutic targets. Microproteins, small proteins of ≤100–150 amino acids, have recently emerged as important regulators of metabolism. Encoded by short open reading frames (sORFs), many of these proteins were historically overlooked due to their small size and misclassification as noncoding RNAs. Advances in genomics and proteomics have revealed that these sORFs can encode functional proteins with critical roles in metabolic pathways. In this review, we highlight the microproteins involved in energy metabolism, mitochondrial function, and nutrient signaling. We discuss their emerging roles in the pathogenesis of NCDs and explore their potential as novel therapeutic targets. As microprotein biology continues to evolve, these small but powerful regulators may offer new strategies for treating metabolic dysfunction and reducing the global burden of NCDs.

## 1. Introduction

Metabolism refers to the body’s ability to break down and process macromolecules such as carbohydrates, fats, and proteins. It comprises numerous complex pathways that convert these macromolecules into energy required for maintaining bodily functions. These pathways are in constant flux, responding to an organism’s nutritional state and energy demands. Any disruption to these complex pathways can result in metabolic dysfunction [1].

The dysregulation of metabolic pathways contributes to the development of noncommunicable diseases (NCDs), including obesity, type 2 diabetes, cancer, and fatty liver disease, among others [2]. According to the Centers for Disease Control and Prevention (CDC), 6 in 10 Americans have been diagnosed with a NCD, and this number continues to rise. NCDs account for approximately 90% of healthcare expenditures in the United States, with costs increasing due to sedentary lifestyles and nutrient-poor diets [3]. Although therapeutic regiments exist for these conditions, NCDs such as heart disease, cancer, diabetes, and liver disease remain leading causes of death in the United States. As a result, continued research into novel therapeutic targets is essential to improving patient outcomes and reducing the health risks associated with NCDs [3].

Microproteins are a class of small proteins that have historically been overlooked due to their small size, often being misannotated as noncoding RNAs or entirely excluded from genome annotations. Microproteins are encoded by short open reading frames (sORFs) and are loosely defined as proteins that are ≤100–150 amino acids in length. Historically, protein identification methods required a minimum ORF length of 300 base pairs, inadvertently excluding many microproteins from detection and annotation. However, recent advancements in ribosome profiling, proteomic techniques, and biochemical methods have revealed that microproteins play critical roles in various cellular processes including calcium cycling, oxidative phosphorylation, and cellular stress signaling.

Microproteins can function independently, but they frequently interact with larger protein partners or integrate into multi-protein complexes to modulate their activity. In addition to acting as primary effectors, microproteins often fine-tune the function of their binding partners by enhancing, inhibiting, or stabilizing their activity. Identifying these interaction partners is key to understanding a microprotein’s function, as they often exert regulatory effects on well-characterized proteins with established roles. Once these interactions are determined, further studies can delineate the precise mechanisms by which microproteins influence cellular processes [4,5,6,7,8,9,10].

As an emerging field, microprotein research has uncovered diverse functional roles in diseases such as cancer, Alzheimer’s disease, and cardiovascular diseases. Many microproteins have been implicated in metabolic regulation, yet their precise mechanisms within metabolic pathways remain underexplored. Some microproteins, such as Dwarf open reading frame (DWORF), are already being investigated as therapeutic targets. These findings highlight not only the biological significance of microproteins but also their potential for clinical applications [6,7,11,12,13]. This review will examine the current field of microprotein research with a primary focus on their proposed or established roles in metabolism.

## 2. Microproteins in Metabolism

Metabolism is tightly regulated by complex networks of proteins that control energy production, nutrient utilization, and cellular homeostasis. While traditionally studied enzymes and signaling proteins have been well-characterized in these processes, recent discoveries have highlighted microproteins as emerging regulators of metabolic pathways. Microproteins influence key aspects of metabolism by modulating enzymatic activity, interacting with metabolic sensors, and altering gene expression. Despite their small size, some microproteins have been implicated in critical metabolic functions, including glucose homeostasis, fatty acid metabolism, oxidative phosphorylation, and lipid regulation. In this section, we explore the current understanding of microprotein involvement in metabolism, highlighting their diverse roles and potential as therapeutic targets.

### 2.1. Glucose Metabolism

**Figure 1:**
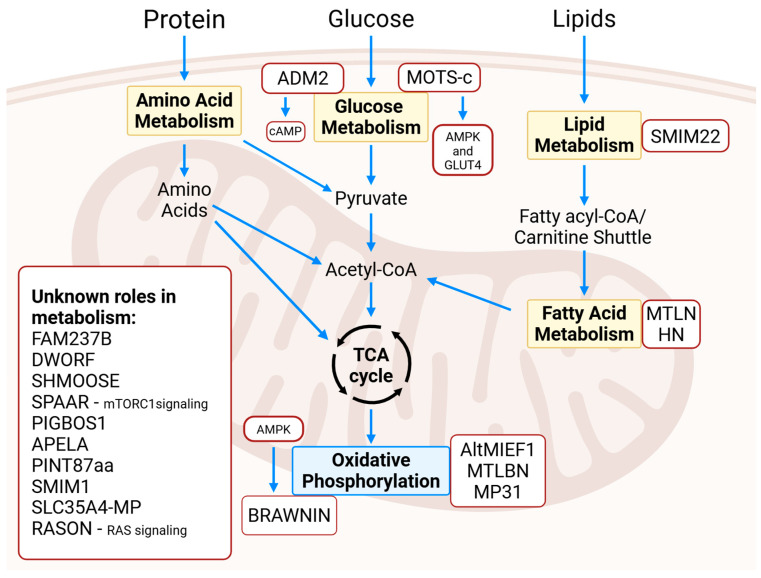
Pathways in which microproteins function in metabolism.

Glucose metabolism is a key component of cellular energy production, involving the breakdown of glucose and related intermediates into substrates that can fuel the tricarboxylic acid (TCA) cycle. This cycle generates adenosine triphosphate (ATP), which is the primary energy currency of the cell [14]. The dysregulation of glucose metabolism contributes to the development of NCDs, including diabetes mellitus, cancer, and cardiovascular disease. A comprehensive understanding of glucose metabolic pathways is therefore essential for identifying new therapeutic targets aimed at reducing risk factors and morbidity associated with these conditions [15]. Emerging evidence suggests that microproteins play both positive and negative regulatory roles in glucose metabolism and energy homeostasis. In this section, we summarize the current literature on microproteins involved in glucose metabolism, highlighting their diverse functions and potential implications in health and disease (Figure 1).

#### 2.1.1. Mitochondrial Opening Reading Frame of the 12S Ribosomal RNA Type-c

Mitochondrial open reading frame of the 12S ribosomal RNA type-c (MOTS-c) is a 16 amino acid microprotein that has been detected in plasma and skeletal muscle. Although encoded in the mitochondrial genome, MOTS-c can translocate to the nucleus in response to cellular stimuli, where it regulates gene expression, particularly metabolic genes [16,17,18]. MOTS-c plays a key role in glucose metabolism by activating AMP-activated protein kinase (AMPK) and glucose transporter 4 (GLUT4), both of which are essential for glucose uptake and utilization. Studies have shown that MOTS-c promotes glucose consumption rather than storage as evidenced by decreased glucose levels, reduced lactate accumulation, and the increased expression of glycolytic enzymes [18]. In wild-type mice, the administration of exogenous MOTS-c enhanced glucose clearance and improved insulin sensitivity [16,18].

The metabolic benefits of MOTS-c also extend to disease models. In diet-induced obesity mouse models, exogenous MOTS-c treatment prevented obesity, reduced hyperinsulinemia, and increased AMPK and GLUT4 activation in skeletal muscle. Additionally, treated mice exhibited increased thermogenesis, suggesting increased energy expenditure over storage, leading to a leaner phenotype even on a high-fat diet [18]. Further studies have shown that MOTS-c modulates immune function, as treatment in autoimmune-induced diabetes decreased CD4+ T cell activation and reduced inflammatory markers such as interferon gamma (IFNγ) [17]. These effects are linked to MOTS-c’s regulation of the mammalian target of rapamycin complex 1 (mTORC1), further supporting its role in insulin regulation in autoimmune-induced diabetes [17,19]. Overall, MOTS-c is a critical metabolic regulator with therapeutic potential in metabolic disorders such as obesity, diabetes, and immune-related metabolic dysfunction [16,18].

#### 2.1.2. Adrenomedullin-2

Adrenomedullin-2 (ADM2, or AM2) is a 148 amino acid microprotein expressed in the pancreas, thyroid, kidney, and liver. While its metabolic functions are not fully understood, ADM2 has been shown to stimulate cyclic AMP (cAMP) release from platelets, contributing to glucose regulation. Additionally, ADM2 is thought to provide protection against oxidative stress and hypoxia, suggesting broader metabolic and homeostatic roles [20,21]. Interestingly, circulating ADM2 levels are elevated in patients with both thyroid cancer and type 2 diabetes compared to those with thyroid cancer alone [22]. Furthermore, metabolic stressors were shown to increase ADM2 expression in a murine thyroid cancer mouse model, and elevated circulating ADM2 was associated with higher body mass index and fasting glucose levels, implicating ADM2 in the metabolic dysregulation observed in thyroid cancer [22]. Further investigation into ADM2′s role in metabolism is warranted to clarify its physiological significance and reconcile its seemingly contradictory effects, beneficial in some contexts and detrimental in others, across different NCDs.

These findings highlight the emerging importance of microproteins in glucose metabolism. From MOTS-c’s ability to improve insulin sensitivity and prevent diet-induced obesity to ADM2′s potential role in glucose regulation through cAMP signaling, and its elevated levels in thyroid cancer patients, microproteins offer promising new avenues for therapeutic intervention aimed at maintaining glucose homeostasis and reducing the burden of NCDs.

### 2.2. Fatty Acid Metabolism

Fatty acid metabolism is a highly complex and tightly regulated process essential for energy production, particularly during periods of fasting and intense exercise. The primary pathway, β-oxidation, involves the breakdown of fatty acids into acetyl-CoA subunits, which then enter the TCA cycle to produce ATP (Figure 1) [23]. Fatty acids are derived either from dietary intake or are mobilized from adipose tissue, after which they are transported into the mitochondria to undergo β-oxidation. The excessive intake of saturated fats has been associated with elevated low-density lipoprotein cholesterol (LDL-C), a major contributor to cardiovascular disease and a risk factor for the development of type 2 diabetes [24]. Alterations in fatty acid metabolism have also been linked to cancer progression, influencing both tumor growth and the surrounding microenvironment [25]. Emerging evidence has identified several microproteins that play regulatory roles in fatty acid metabolism, influencing lipid utilization, energy homeostasis, and metabolic health. This section highlights the key microproteins involved in fatty acid metabolism and their implications in metabolic regulation.

#### 2.2.1. Mitoregulin

Mitoregulin (MTLN, or LEMP, MOXI, MPM, and SMIM37) is a 56 amino acid transmembrane microprotein that is ubiquitously expressed and particularly enriched in cardiac and skeletal muscle. MTLN has been independently identified and characterized by several research groups [26,27,28,29,30,31,32]. While accumulating evidence suggests that MTLN plays a role in mitochondrial fatty acid β-oxidation, its exact molecular function remains incompletely understood. Beyond fatty acid metabolism, MTLN has been implicated in additional aspects of mitochondrial physiology, including the regulation of oxidative phosphorylation (OXPHOS), reactive oxygen species (ROS) levels, and mitochondrial membrane potential [26].

However, inconsistencies in the literature, particularly regarding MTLN’s sub-mitochondrial localization and interacting partners, have made it challenging to define a unifying functional model. MTLN has been variably localized to both the outer and inner mitochondrial membranes. Reported interacting proteins include the following: outer membrane components such as CYB5R3 [28], CYB5B, and CPT1 [32]; inner membrane proteins including NDUFA7 [29] and ATP5B [30]; and matrix-facing components such as HADHA and HADHB [27,30].

Functional studies of MTLN-deficient models have produced a wide range of phenotypes, including impaired fatty acid oxidation, disrupted calcium handling, smaller skeletal muscle fibers, and conflicting effects on mitochondrial respiration, with some studies reporting reduced respiratory capacity, while others observed enhanced activity [26,27,28,29,30,31,32]. Together, these conflicting results underscore the complexity of MTLN’s biology. While compelling evidence supports a role in mitochondrial and metabolic function, the exact mechanisms and contexts in which MTLN operates remain to be fully clarified. Further studies are needed to reconcile these discrepancies and define MTLN’s physiological and pathological relevance.

#### 2.2.2. Humanin

Humanin (HN) is a 24 amino acid microprotein expressed in various tissues including male reproductive organs, eyes, liver, kidney, and both cardiac and skeletal muscle. HN was originally identified in neuronal cells from the occipital lobe of a patient with autopsy-confirmed Alzheimer’s disease and was found to protect neuronal cell lines from cell death [33]. In addition to its neuroprotective functions, HN has been studied in human endothelial cells treated with free fatty acids (FFAs) to model metabolic stress. FFAs induce an increase in lactate dehydrogenase (LDH), an enzyme involved in converting lactate to pyruvate during anabolic metabolism. Elevated LDH levels are associated with conditions such as muscle injury, cardiovascular disease, and liver dysfunction. Treatment with HN significantly reduced LDH levels in a dose-dependent manner, suggesting a protective role against lipid-induced metabolic stress. This effect was linked to HN’s ability to suppress oxidative stress by inhibiting NADPH oxidase 2 (NOX2) signaling. Furthermore, HN treatment decreased the expression of inflammatory markers IL-1β and IL-18, indicating its potential role in mitigating inflammation and metabolic dysfunction in endothelial cells [34].

These studies collectively underscore the emerging significance of microproteins as regulators of fatty acid metabolism and mitochondrial function, particularly in the context of metabolic stress and disease. Microproteins such as MTLN and HN illustrate the diversity of mechanisms through which these small peptides influence lipid utilization, oxidative stress, calcium handling, mitochondrial respiration, and inflammatory signaling. Importantly, conflicting findings across models focused on defining the function of MTLN emphasize the complexity and context-dependent nature of microprotein function, highlighting the need for further mechanistic studies. Continued investigation into these and other microproteins will be essential to fully understand their roles in maintaining metabolic homeostasis and their potential as therapeutic targets for NCDs.

### 2.3. Lipid Metabolism

Lipid metabolism plays a central role in both the breakdown of dietary fats and the synthesis of endogenous lipids required for essential cellular functions, such as membrane formation, energy storage, and signaling. Among the most well-characterized pathways are those regulating the metabolism of triglycerides and cholesterol [35]. Dysregulation of these pathways is strongly associated with NCD comorbidities, including obesity, hyperlipidemia, type 2 diabetes, and cardiovascular disease. Although pharmacological treatments—most notably statins—are widely used to manage dyslipidemia, the global burden of lipid-related disorders remains high and continues to increase [36]. In this section, we focus on the emerging role of the microprotein SMIM22 in lipid metabolism and its potential contribution to metabolic dysregulation in NCDs.

#### Small Integral Membrane Protein 22

Small integral membrane protein 22 (SMIM22, or CASIMO1) is an 83 amino acid microprotein predominantly expressed in the stomach and digestive tract. While SMIM22 has been primarily studied in the context of cancer tumorigenesis, recent research suggests that it may also contribute to lipid metabolism [37,38]. SMIM22 is predicted to be a component of exosomes and interact with squalene epoxidase (SQLE), a key enzyme in cholesterol biosynthesis. The overexpression of SMIM22 in vitro led to a significant increase in lipid droplet accumulation, suggesting a role in lipid storage. Similarly, cells with normal SMIM22 levels exposed to oleic acid, a stabilizer of SQLE, also exhibited increased lipid droplet formation. When both SMIM22 overexpression and oleic acid treatment were combined, lipid accumulation was further exacerbated, reinforcing SMIM22′s role in lipid droplet biogenesis and cholesterol metabolism [38]. Although the mechanisms by which SMIM22 regulates lipid biogenesis remain unclear, its upregulation in cancers such as non-small-cell lung cancer and hepatocellular carcinoma raises the possibility that it may contribute to tumor progression through effects on lipid metabolism. In these contexts, SMIM22 has been shown to enhance tumor cell proliferation, supporting a potential dual role in both metabolic regulation and cancer progression [37,39].

These findings highlight a novel role for SMIM22 in lipid homeostasis, particularly in promoting lipid droplet formation and potentially influencing cholesterol metabolism. While its precise molecular function remains to be fully defined, the ability of SMIM22 to modulate lipid storage pathways points to broader metabolic implications. Further studies are warranted to determine the physiological relevance of SMIM22 in vivo and its potential involvement in the pathogenesis of lipid-associated metabolic disorders.

### 2.4. Oxidative Phosphorylation

Oxidative phosphorylation is a critical component of cellular metabolism, responsible for generating the majority of ATP used by cells for energy. It relies on the transfer of electrons from NADH and FADH_2_, primarily generated by the TCA cycle, to the electron transport chain (ETC) located in the inner mitochondrial membrane. This process drives the production of ATP through chemiosmotic coupling via ATP synthase. Because oxidative phosphorylation is central to energy homeostasis, tissues with high metabolic demand, such as the heart, brain, and skeletal muscle, are particularly vulnerable to mitochondrial dysfunction. Impairment of this pathway has been linked to a range of NCDs, including cardiovascular disease, where energy deficiency in cardiomyocytes contributes to disease progression. In this section, we highlight mitochondrial microproteins that modulate oxidative phosphorylation and discuss their potential roles in regulating mitochondrial efficiency and metabolic health.

#### 2.4.1. Mitochondrial Ribosome and Complex I Assembly Factor

Mitochondrial ribosome and complex I assembly factor (AltMIEF1, or AltMiD51) is a 70 amino acid microprotein that localizes to the mitochondrial matrix. Its specific tissue expression has not been well-characterized. This microprotein has been classified as an “alternative” protein by Samandi et al. [40] as it originates from the 5′ untranslated region (UTR) of mitochondrial elongation factor 1 (MIEF1). Given its genomic context, AltMIEF1 is believed to function in a manner similar to its host gene, MIEF1, which regulates mitochondrial fission. Recent studies have also implicated AltMIEF1 in mitochondrial ribosome function [40,41], identifying it as a ribosome-associated protein likely involved in ribosome assembly and intramitochondrial protein synthesis. Functional knockouts of AltMIEF1 resulted in a decrease in oxidative phosphorylation, underscoring its importance in mitochondrial metabolism [41]. These findings highlight AltMIEF1 as a key regulator of mitochondrial dynamics and energy production.

#### 2.4.2. Ubiquinol–cytochrome c Reductase Complex Assembly Factor 6

Ubiquinol–cytochrome c reductase complex assembly factor 6 (UQCC6, or BRAWNIN, BR, C12orf73) is a 71 amino acid microprotein localized to the inner mitochondrial membrane. BRAWNIN is ubiquitously expressed but is most highly enriched in skeletal muscle and brown adipose tissue, both of which are metabolically active tissues [42]. The overexpression of BRAWNIN has been shown to enhance OXPHOS, indicating a role in energy metabolism. Its expression is upregulated upon AMP-activated protein kinase (AMPK) activation, suggesting a link between BRAWNIN and cellular energy sensing. The knockdown of BRAWNIN in glioblastoma cells led to reduced ATP levels and decreased AMPK activity, reinforcing its role in energy homeostasis. Co-immunoprecipitation studies further demonstrated that BRAWNIN interacts with mitochondrial complex III subunits, supporting its function in facilitating oxidative phosphorylation and mitochondrial energy production [42].

#### 2.4.3. Mitolamban

Mitolamban (Mtlbn), also known as transmembrane microprotein 1 (STMP1) and mitochondrial micropeptide 47 (Mm47), is a 47 amino acid microprotein localized to the mitochondria [43,44]. Mtlbn is ubiquitously expressed, with its highest expression in skeletal and cardiac muscle, bone marrow, and the esophagus. Mtlbn localizes to the inner mitochondrial membrane, where its transmembrane domain suggests a functional role in oxidative phosphorylation [43,45]. Proteomic analyses have identified Mtlbn in association with several components of the electron transport chain, particularly complex III in cardiac tissue. In cardiac-specific Mtlbn knockout mice, complex III enzymatic activity was significantly reduced despite the absence of overt cardiac dysfunction. Conversely, cardiac-specific overexpression of Mtlbn resulted in a cardiomyopathy phenotype marked by increased oxidative stress, although complex III activity was unaffected under these conditions [43]. Additionally, Mtlbn has been shown to enhance complex IV activity [46] and activate dynamin-related protein 1 (DRP1), promoting mitochondrial fission and enhancing tumor cell migration [47,48].

Beyond bioenergetics, Mtlbn also contributes to immune and inflammatory responses. It is required for activation of the NLRP3 inflammasome [49] and has been implicated in modulating microglial inflammatory signaling through the regulation of mitochondrial function [50]. Together, these findings position Mtlbn as a multifunctional microprotein involved in mitochondrial respiration, redox balance, immune signaling, and cellular stress adaptation.

#### 2.4.4. Micropeptide 31

Micropeptide 31 (MP31) is a mitochondria-localized peptide encoded by an upstream open reading frame (uORF) within the 5′ untranslated region of the PTEN gene. Acting as a metabolic regulator, MP31 restricts the conversion of lactate to pyruvate by competing with mitochondrial lactate dehydrogenase (mLDH) for NAD^+^, thereby dampening mitochondrial oxidative metabolism [51]. Loss of MP31 leads to enhanced lactate utilization, increased oxidative phosphorylation, and metabolic reprogramming that supports tumorigenesis. In mouse models, astrocyte-specific deletion of the MP31 homolog initiates glioma development, supporting its role as a tumor suppressor. Importantly, systemic delivery of recombinant MP31 suppresses glioblastoma growth in vivo and crosses the blood–brain barrier without neurotoxicity, underscoring its potential as a novel therapeutic strategy targeting cancer metabolism. Although MP31 has not yet been studied in the context of other NCDs, its ability to modulate oxidative phosphorylation highlights the need for further investigation into its potential roles beyond cancer.

Oxidative phosphorylation is essential for cellular energy production, and its disruption has been linked to the pathogenesis of various NCDs, with several microproteins emerging as key regulators of this pathway. AltMEIF1 has been shown to influence mitochondrial protein synthesis, indirectly supporting oxidative phosphorylation. BRAWNIN and Mitolamban (Mtlbn) both contribute to the regulation of complex III activity, underscoring their importance in maintaining efficient electron transport and ATP generation. Notably, Mtlbn has also been associated with cardiac dysfunction and may play a role in the development of heart failure. MP31, which competes with mLDH for NAD^+^ to limit lactate-driven oxidative metabolism, further illustrates how microproteins can fine-tune mitochondrial function in a disease-relevant context. Together, these findings highlight microproteins as potential therapeutic targets for mitochondrial dysfunction, though further studies are needed to fully elucidate the mechanisms by which they regulate oxidative phosphorylation.

### 2.5. Erythrocyte Metabolism

Erythrocytes, or red blood cells, are vital for transporting oxygen from the lungs to tissues and returning carbon dioxide for exhalation. To carry out these functions, erythrocytes rely exclusively on ATP produced through glycolysis, as they lack mitochondria [52]. Proper red blood cell function is essential for systemic oxygen delivery and, by extension, the health of all organ systems. In this section, we highlight the role of a microprotein expressed in erythrocytes and its potential link to red blood cell metabolism.

#### Small Integral Membrane Protein 1

Small integral membrane protein 1 (SMIM1) is a 78 amino acid microprotein expressed in male reproductive tissues, skeletal muscle, bone marrow, and red blood cells. SMIM1 was identified through studies on blood group antigens, specifically the Vel antigen. While most individuals express Vel on their red blood cells, a small subset of the population lacks this antigen. Vel-negative individuals can develop antibodies against Vel, known as anti-Vel, which can lead to hemolytic transfusion reactions, an immune-mediated destruction of transfused red blood cells [53,54].

The clinical relevance of anti-Vel reactions spurred efforts to uncover the molecular identity of the Vel antigen. Traditional serological methods for Vel antigen detection have limitations, making molecular identification crucial. Despite numerous attempts, the molecular basis of the Vel blood group remained elusive until Ballif et al. [53] successfully purified the Vel antigen and identified SMIM1 as its carrier using mass spectrometry-based sequencing. Their findings revealed that a deletion in *SMIM1* is the primary cause of the Vel^−^ blood type.

While SMIM1 is best known for its role in blood group antigenicity, red blood cells are metabolically active, performing essential functions such as ATP generation, redox balance, and maintenance of membrane integrity. Although SMIM1′s direct involvement in erythrocyte metabolism has not been firmly established, its contribution to membrane structure and stability suggests a possible influence on metabolic efficiency and cellular homeostasis. Further research is warranted to investigate the potential metabolic functions of SMIM1 in red blood cells.

### 2.6. General Metabolism

While some microproteins have defined roles in specific pathways like glucose or lipid metabolism, others exert broader effects that contribute to overall metabolic homeostasis. Despite their small size, these proteins can interact with major metabolic regulators, affect mitochondrial function, and modulate cellular responses to environmental changes. This section highlights microproteins with diverse roles in metabolism, including those involved in appetite regulation, calcium signaling, and mitochondrial dynamics, underscoring their potential impact on metabolic health.

#### 2.6.1. Small Regulatory Polypeptide of Amino Acid Sequences

Small regulatory polypeptide of amino acid sequences (SPAAR, SPAR) is a 90 amino acid microprotein encoded by the long noncoding RNA LINC00961. SPAAR is ubiquitously expressed but enriched in adipose tissue, placenta, lung, skeletal muscle, and cardiac muscle. SPAAR has been shown to localize to late endosomes and lysosomes, where it complexes with the v-ATPase proton pump [55]. SPAAR regulates mTORC1 recruitment to the lysosome, with overexpression inhibiting its localization and activation. In skeletal muscle, SPAAR expression is downregulated following injury, leading to increased mTORC1 activation and enhanced muscle regeneration [55,56]. While the role of the SPAAR microprotein in cancer remains unexplored, recent studies have implicated the LINC00961 transcript itself in tumor suppression in non-small-cell lung cancer (NSCLC). Specifically, LINC00961 is significantly downregulated in NSCLC tissues, with reduced expression associated with advanced clinical stage, metastasis, and poor prognosis. Functional studies demonstrated that LINC00961 overexpression inhibits NSCLC cell invasion and metastasis, likely through mechanisms independent of SPAAR translation, possibly involving epigenetic regulation and β-catenin signaling [57]. These findings suggest that both SPAAR and its parent lncRNA may have distinct biological functions, and further investigation is warranted to determine whether SPAAR also contributes to cancer metabolism or mTORC1 signaling in disease contexts beyond muscle.

#### 2.6.2. PIGB Opposite Protein 1

PIGB opposite protein 1 (PIGBOS1) is a 54 amino acid microprotein that is ubiquitously expressed and enriched in the pancreas, thyroid, and spleen. It localizes to the outer mitochondrial membrane and interacts with chloride channel CLIC-1 (CLCC1), an endoplasmic reticulum (ER)-localized protein. This interaction is believed to occur between the cytoplasmic-facing C-terminal region of PIGBOS1 and the outer surface of the ER membrane [58]. Studies have demonstrated that PIGBOS1 plays a role in the unfolded protein response (UPR), a cellular stress response activated under ER stress conditions. Cells lacking PIGBOS1 exhibit a lower threshold for UPR activation, indicating that PIGBOS1 is involved in maintaining ER homeostasis. Given the established links between ER stress and metabolic disorders such as diabetes and obesity, PIGBOS1 likely contributes to metabolic regulation, though further research is needed to clarify its mechanisms [59,60].

#### 2.6.3. Small Human Mitochondrial ORF over Serine tRNA

Small human mitochondrial ORF over serine tRNA (SHMOOSE) is a 58 amino acid mitochondrial microprotein that is highly expressed in the brain. SHMOOSE was initially discovered in studies investigating mitochondrial single nucleotide polymorphisms associated with Alzheimer’s disease. It has been shown that SHMOOSE interacts with mitofilin, a key protein located between the inner and outer mitochondrial membranes that is part of a complex responsible for maintaining mitochondrial function [61,62]. Although current research primarily focuses on SHMOOSE’s role in neurodegenerative disease, its involvement in mitochondrial function suggests a broader impact on neuronal metabolism. Given the importance of mitochondrial efficiency in neuroenergetics, SHMOOSE may influence metabolic processes beyond neurodegeneration, but further studies are needed to define its specific metabolic roles [62].

#### 2.6.4. Dwarf Open Reading Frame

Dwarf open reading frame (DWORF, or STRIT1) is a 35 amino acid microprotein that is highly expressed in cardiac and slow (oxidative) skeletal muscle fibers [63]. DWORF localizes to the sarcoplasmic reticulum (SR) membrane, where it directly binds the Sarco/Endoplasmic Reticulum Calcium-ATPase (SERCA), an ATP-dependent pump that transports calcium from the cytoplasm into the SR to induce muscle relaxation. DWORF functions by competitively binding to SERCA, displacing its inhibitors—phospholamban (PLN), myoregulin (MLN), sarcolipin (SLN), and another-regulin (ALN)—to enhance calcium uptake [6,7,10,11,63]. This regulation of calcium cycling not only supports cardiac and skeletal muscle function but also influences mitochondrial calcium homeostasis, which is tightly linked to cellular energy demand and metabolism. DWORF is downregulated in mice and human hearts in heart failure as well as heart diseases such as dilated cardiomyopathy [11,12,63].

#### 2.6.5. Apelin Receptor Early Endogenous Ligand

Apelin receptor early endogenous ligand (APELA, ELABELA, or TODDLER) is a 54 amino acid microprotein that functions as a hormone in kidney, male reproductive tissues, blood vessels, and embryonic and induced adult pluripotent stem cells [64]. APELA activates the G-protein coupled receptor, apelin, leading to decreased cAMP production and increased intracellular calcium signaling [65]. Although APELA’s metabolic functions remain unclear, its interactions with apelin, cAMP, and calcium suggest that it may influence metabolic pathways indirectly. APELA expression has been detected in several types of cancer; however, its potential roles in tumor metabolism and the underlying mechanisms remain largely unexplored [66,67,68,69]. Further studies are required to determine whether APELA contributes to cellular energy balance and metabolic adaptation [64,65].

#### 2.6.6. PINT87aa

PINT87aa is an 87 amino acid microprotein encoded from circular RNA originating from exon 2 of LINC-PINT, known as circPINTexon2. Unlike its host gene, which functions as a long noncoding RNA, PINT87aa has been identified as an independent regulatory microprotein involved in cell proliferation and tumorigenesis [70]. While no direct metabolic role has been reported, PINT87aa interacts with polymerase-associated factor 1 (PAF1), anchoring it to facilitate RNA polymerase II activation and mRNA translation [70]. This transcriptional regulation may have downstream effects on metabolic pathways, though further research is needed to clarify its specific role in cellular metabolism.

#### 2.6.7. SLC35A4 Upstream Open Reading Frame Protein

SLC35A4 upstream open reading frame protein (SLC35A4-MP), also referred to as SLC35A4 uORF, is a 103 amino acid microprotein that is ubiquitously expressed and highly enriched in skeletal muscle. It is encoded by an upstream open reading frame (uORF) of SLC35A4 and localizes to the inner mitochondrial membrane [71]. Knockout studies in HEK293T cells revealed that loss of SLC35A4-MP resulted in reduced cellular respiration without affecting oxidative phosphorylation-related proteins. Conversely, overexpression of SLC35A4-MP led to a significant increase in cellular respiration, indicating its involvement in mitochondrial energy metabolism. However, further studies are needed to determine its precise molecular function.

#### 2.6.8. Family with Sequencing Similarity 237 Member B

Family with sequencing similarity 237 member B (FAM237B) is a 139 amino acid microprotein expressed in the brain, eyes, adipose tissue, and reproductive organs. This microprotein was recently discovered by the ribosomal profiling of wild-type mouse white and brown adipose tissues, but its function remains largely unknown. Martinez et al. [72] identified Gm8773, a mouse homolog to FAM237B, as a secreted microprotein with a role in regulating feeding signals by increasing appetite. While this suggests a role in metabolism, the exact pathways through which FAM237B influences metabolic processes remain to be elucidated.

#### 2.6.9. RAS-ON

RAS-ON, or RASON, is a 108 amino acid microprotein encoded by the long noncoding RNA LINC00673 and is highly expressed in tumors harboring oncogenic KRAS mutations, including pancreatic ductal adenocarcinoma [73]. RASON enhances RAS pathway activity by directly binding to mutant KRAS proteins and inhibiting their GTP hydrolysis, thereby maintaining them in a hyperactive, GTP-bound state. Loss of RASON impairs tumor growth in vivo and sensitizes KRAS-mutant pancreatic cancer cells to EGFR inhibition, highlighting its function as a key modulator of oncogenic signaling. Given that KRAS mutations are known to reprogram glucose and glutamine metabolism to support tumor growth [74], RASON may indirectly influence these metabolic pathways by sustaining oncogenic KRAS activity. Although RASON has not yet been directly linked to metabolic regulation, RAS signaling broadly impacts cellular metabolism, suggesting a possible role for RASON in metabolic reprogramming. Given the involvement of RAS pathways in other noncommunicable diseases, such as cardiovascular disorders, RASON may also have broader relevance beyond cancer, and this warrants further investigation

Together, these microproteins illustrate the diverse and often unexpected ways in which small peptides can influence metabolic regulation, from mitochondrial energy production and calcium handling to appetite signaling and cellular stress responses, highlighting their potential as emerging targets in metabolic research.

## 3. Conclusions

**Figure 2:**
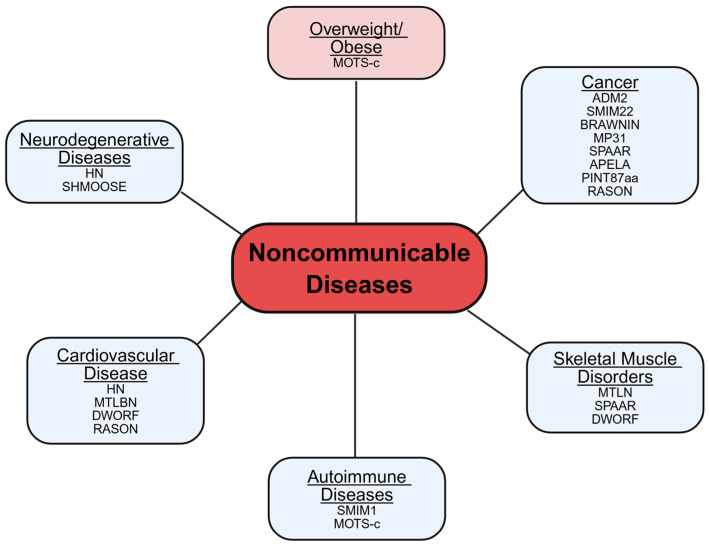
Metabolic microproteins in noncommunicable diseases. Microproteins implicated in noncommunicable diseases are shown in blue, while those associated with conditions that contribute to noncommunicable disease development are shown in pink.

The global burden of NCDs continues to rise despite the widespread use of pharmacological therapies. There is a critical need for novel treatment strategies that not only manage symptoms but also address the underlying mechanisms driving disease progression. One emerging area of promise is microprotein biology, a field propelled by advances in sequencing and proteomics that have revealed that many transcripts previously classified as noncoding RNAs in fact encode small, functional proteins. Although our understanding of microproteins is still evolving, mounting evidence suggests that they play integral roles in diverse cellular processes, with broad implications for health and disease, including NCDs (Figure 2).

To facilitate the discovery and functional study of microproteins, researchers have developed innovative molecular tools. A particularly powerful approach is endogenous epitope tagging, where a short peptide tag is inserted in-frame at the native genomic locus of the microprotein gene. This enables detection of the microprotein under its natural regulatory context using techniques such as immunostaining, Western blotting, and immunoprecipitation. Endogenous tagging is especially valuable for studying small or transmembrane microproteins, which often lack sufficient epitopes for traditional antibody-based detection. These tools are instrumental in determining subcellular localization, protein–protein interactions, and physiological function, paving the way for deeper mechanistic insights into microprotein biology. 

The therapeutic potential of microproteins is already beginning to emerge. For example, DWORF, a microprotein that enhances calcium cycling in muscle cells, is under investigation for its role in cardiomyopathy and muscular dystrophy [12,13]. As the functional relevance of more microproteins becomes clear, they may serve as novel therapeutic targets or even be harnessed as therapeutic agents themselves.

The growing recognition of microproteins as key regulators of cellular function is reshaping our understanding of molecular biology. Unlike traditional proteins, microproteins have often been overlooked due to their small size, noncanonical coding sequences, and lack of annotation in reference genomes. However, their discovery is revealing a previously unappreciated layer of biological complexity. Integrating microproteins into existing biological frameworks will require multidisciplinary collaboration across genomics, proteomics, bioinformatics, and structural biology. Understanding how microproteins intersect with established signaling pathways and metabolic networks could unlock new diagnostic and therapeutic opportunities, particularly in diseases where current treatments fall short (Table 1). As research in this field accelerates, continued investment in microprotein biology will be essential not only for advancing fundamental knowledge but also for driving innovation in precision medicine, drug development, and disease prevention.

## Figures and Tables

**Table 1:** Information on microproteins in metabolism.

Human Gene	Microprotein Size (Amino Acids)	Tissue Expression	Mouse Homolog Gene	Reference
Adrenomedullin-2 (ADM2, AM2)	148	Pancreas, thyroid grand, kidney, liver	Adm2, Am2	[20,21,22]
Apelin receptor early endogenous ligand (APELA, ELABELA, TODDLER)	54	Kidney, male reproductive tissues, pluripotent stem cells, blood vessels	Apela, Ela, Ende, Gm10664, Tdl	[64,65,66,67,68,69]
Dwarf opening reading frame (DWORF, STRIT1)	35	Skeletal and cardiac muscle	Dworf, Strit1	[11,12,63]
Family with sequence similarity 237 member B (FAM237B)	139	Brain, eye, adipose tissue, reproductive organs	Fam237b, Gm8773, EG667705	[72]
Humanin (HN)	24	Male reproductive tissues, eye, cardiac and skeletal muscle, liver, kidney	Mtrnr2l7, Gm20594	[33,34]
Microprotein 31 (MP31)	31	Brain, specifically studied in glioblastomas	MP35	[51]
Mitochondrial ribosome and complex I assembly factor (AltMIEF1, AltMiD51)	70	N/a	N/a	[40,41]
Mitochondrial opening reading frame of the 12S ribosomal RNA type-c (MOTS-c)	16	Plasma and skeletal muscle	N/a	[16,17,18,19]
Mitolamban (MTLBN, STMP1, C7orf73)	47	Ubiquitous, highest in skeletal and cardiac muscle	Mtlbn, Stmp1, Mm47	[43,44,45,46,49]
Mitoregulin (MTLN, LEMP, MOXI, MPM, SMIM37)	56	Ubiquitous, highest in skeletal and cardiac muscle	Mtln, Lemp, Moxi, Mpm, Smim37	[26,27,28,29,30,31,32]
PIGB opposite protein 1 (PIGBOS1)	54	Ubiquitous, highest in pancreas, thyroid, and spleen	N/a	[58]
PINT87aa	87	Brain, liver, kidney, stomach, breast tissue, intestine, and thyroid	N/a	[70]
RAS-ON (RASON)	108	Tumors	N/a	[73]
SLC35A4 upstream open reading frame protein (SLC35A4-MP)	103	Ubiquitous, highest in skeletal muscle	N/a	[71]
Small human mitochondrial ORF over serine tRNA (SHMOOSE)	58	Brain	N/a	[62]
Small integral membrane protein 1 (SMIM1)	78	Male reproductive tissues, skeletal muscle, bone marrow, red blood cells	Smim1	[53,54]
Small integral membrane protein 22 (SMIM22, CASIMO1)	83	Stomach, digestive tract	Smim22, Gm5480	[37,38,39]
Small regulatory polypeptide of amino acid sequences (SPAAR, SPAR)	90	Ubiquitously expressed, most highly expressed in adipose tissue, placenta, lung, and skeletal and cardiac muscle	Spaar, Spar	[55,56,57]
Ubiquinol–cytochrome c reductase complex assembly factor 6 (UQCC6, BR, BRAWNIN, C12orf73)	71	Ubiquitous, highest in skeletal muscle and brown adipose tissue	Uqcc6, Br, Brawnin	[42]

## Data Availability

No new data were created or analyzed in this study.
